# Entwicklung einer Agenda für Rehabilitationsforschung in der österreichischen Pensionsversicherung

**DOI:** 10.1055/a-2563-6776

**Published:** 2025-06-10

**Authors:** Doreen Stöhr, Martin Matzka, Monika Mustak-Blagusz, David Felder

**Affiliations:** 1 Forschung, Innovation und medizinische Leistungsentwicklung, Pensionsversicherungsanstalt, Wien, Österreich; 2 Chefärztlicher Bereich, Pensionsversicherungsanstalt, Wien, Österreich

**Keywords:** Forschungsagenda, Rehabilitationsforschung, Delphi-Befragung, Interviews, Multiperspektivisch, research agenda, rehabilitation research, delphi survey, interviews, multiperspective

## Abstract

**Ziel der Studie:**

Forschungsagenden strukturieren Bereiche, Themen und Inhalte eines bestimmten Interessengebietes, in denen bzw. zu denen besonderer Forschungsbedarf besteht. Ziel war es, eine organisationsspezifische Forschungsagenda für die Rehabilitationsforschung in der Pensionsversicherung (PV) zu entwickeln, welche die Erkenntnisinteressen der verschiedenen Akteur*innen in der Rehabilitation widerspiegelt.

**Methodik:**

Die Entwicklung erfolgte multiperspektivisch und multimethodisch anhand eines sequenziellen und iterativen Forschungsdesigns mit qualitativen und quantitativen Anteilen. Es wurden eine narrative Literaturrecherche und problemzentrierte Interviews mit Expert*innen aus der Rehabilitation (Praxis, Verwaltung, Wissenschaft) durchgeführt. Die generierten Daten wurden analysiert, aufbereitet und zusammengeführt. Sie bildeten die Grundlage für eine anschließende zweistufige Delphi-Befragung, in der die Priorität potentieller Forschungsinhalte abgefragt wurden. Die Ergebnisse bildeten die Basis für die Forschungsagenda.

**Ergebnisse:**

Insgesamt wurden 116 konkrete Forschungsinhalte identifiziert, im Zuge der Delphi-Befragung hinsichtlich ihrer Forschungspriorität bewertet und in die Forschungsagenda aufgenommen. Diese konnten in 32 Forschungsthemen, elf Forschungsfeldern und die vier übergeordneten Forschungsbereiche Individuum, Intervention, Institution und Interdisziplinäre Forschung mit schrittweise zunehmendem Abstraktionsgrad zusammengefasst werden.

**Schlussfolgerung:**

Die multimethodische und multiperspektivische Entwicklung der Forschungsagenda ermöglicht eine umfassende Annäherung an die komplexe inhaltliche Vielfalt in der Rehabilitation. Die Forschungsagenda wird dazu beitragen, zukünftige Forschungsaktivitäten in der PV zu strukturieren und an den Bedürfnissen der unterschiedlichen Akteur*innen in der Rehabilitation auszurichten. Ebenso zeigt sie die methodischen, theoretischen und institutionellen Grundlagen der Rehabilitationsforschung auf, um interdisziplinär relevante Forschung betreiben zu können.

## Abstract (en)

**Purpose:**

Research agendas address areas, topics and contents where research is particularly needed. The aim of the study was to develop an organisation-specific research agenda for rehabilitation research in the pension insurance institution (PV), reflecting the research interests of various stakeholders in rehabilitation.

**Methods:**

The study was developed using a sequential, iterative research design with both qualitative and quantitative components. A narrative literature review and in-depth interviews were conducted with experts from the rehabilitation sector (practice, administration and science). The data generated were analysed, processed and consolidated. A subsequent Delphi survey was conducted in two rounds to identify the priority of potential research. The results were used as a basis for the final formulation of the research agenda.

**Results:**

The Delphi survey helped to identify and prioritise a total of 116 concrete research items for inclusion in the research agenda. These were categorised, at progressively higher levels of abstraction, into 32 research topics, eleven research fields and four main research areas namely, Individual, Intervention, Institution and Interdisciplinary research.

**Conclusion:**

The interdisciplinary and multi-perspective development of the research agenda enabled a comprehensive exploration of the complex variety of topics/issues in rehabilitation. The research agenda will help to structure future research activities in PV and align them with the needs of the different stakeholders in rehabilitation. The methodological, theoretical and institutional foundations of rehabilitation research are also adressed to enable interdisciplinary research.

## Einleitung

Angesichts der Vielzahl von Akteur*innen, der Vielfalt der Problemstellungen in der Rehabilitation und den daraus resultierenden Erkenntnisinteressen ist es entscheidend, eine Forschungsagenda zu entwickeln, die die Themenbereiche der Rehaforschung strukturiert und transparent darstellt. Eine Forschungsagenda ist ein strategisches Dokument, das Prioritäten, Ziele und Schwerpunkte für aktuelle und zukünftige Forschungsaktivitäten in einem bestimmten Bereich oder einer Disziplin festlegt. Sie dient der Planung und Steuerung der Forschungsvorhaben und gewährleistet, dass die verfügbaren Ressourcen effizient genutzt werden, um eine kontinuierliche Weiterentwicklung des Forschungsbereichs zu fördern und den wechselseitigen Wissenstransfer zwischen Forschung und Praxis zu unterstützen. Im Bereich der Rehabilitationswissenschaften existieren bereits internationale Forschungsagenden 1 2 , die sich auf spezifische Themenbereiche wie beispielsweise die Physiotherapie 3 4 5 , Onkologie 6 oder Covid-19 7 beziehen. Da für Österreich derzeit keine rehabilitationswissenschaftliche Forschungsagenda vorliegt bzw. auf die spezifische Situation und die spezifischen Erfordernisse der Pensionsversicherung (PV) angepasst werden könnte, hat sich die PV zum Ziel gesetzt, eine eigene organisationsspezifische Forschungsagenda zu entwickeln.

Die PV ist der größte Pensionsversicherungsträger in Österreich und stellt unter anderem Maßnahmen wie medizinische, berufliche und soziale Rehabilitation für Versicherte bereit, um einen Pensionsantritt aus gesundheitlichen Gründen hinauszuzögern bzw. zu verhindern und gesellschaftliche Teilhabe zu fördern bzw. zu ermöglichen. Die medizinischen Maßnahmen der Rehabilitation werden dabei in den 17 von der PV selbst betriebenen Rehabilitationszentren oder in Vertragseinrichtungen durchgeführt. Rehabilitation wird grundsätzlich als multiprofessionelle, multimodale und auf einem bio-psycho-sozialen Gesundheitsverständnis 8 beruhende Intervention verstanden. Dementsprechend besteht ein umfangreicher und vielfältiger Forschungsbedarf sowohl zur Förderung der lokalen Rehabilitationspraxis als auch zur Erweiterung der rehabilitationswissenschaftlichen Wissensbasis. Bis zur Gründung einer dedizierten Abteilung für angewandte Forschung in der PV wurde Rehaforschung vorwiegend individuell in den einzelnen Rehabilitationszentren durchgeführt. Hierzu zählen insbesondere Initiativprojekte von Mitarbeiter*innen und anlassbezogene Kooperationen mit externen Bildungs- und Forschungseinrichtungen im Rahmen wissenschaftlicher Studien und Qualifikationsarbeiten. Mit der Gründung der Forschungsabteilung erweiterte sich der Möglichkeitsraum und der Bedarf einer gründlichen Exploration und Strukturierung des Forschungsgebietes.

Um eine entsprechende Forschungsagenda für die Rehaforschung in der PV zu entwickeln, wurden drei Forschungsfragen formuliert:

Welche Forschungsinhalte werden international in Agenden und Grundlagenwerken zur Rehaforschung beschrieben?
Welche Forschungsinhalte werden von Expert*innen aus Rehawissenschaft und Rehapraxis beschrieben?
Wie bewerten Reha-Expert*innen die Forschungspriorität der identifizierten Forschungsinhalte?

Im Folgenden wird der dreistufige methodische Entwicklungsprozess beschrieben, der eine narrative Literaturrecherche sowie problemzentrierte Interviews und eine Delphi-Befragung von Expert*innen aus dem Rehabereich umfasst und die Grundlage für die vorliegende PV-interne Forschungsagenda geschaffen hat. Die Ergebnisse werden anhand der vier identifizierten Forschungsbereiche mit insgesamt elf Forschungsfeldern und 32 Forschungsthemen dargestellt.

## Methode

Um die Forschungsfragen zu beantworten wurde ein multiperspektivisches, multimethodisches Design mit sequentiellen qualitativen und quantitativen Elementen gewählt. Die Studie wurde bei Clinical Trials registriert (ID: NCT05494892).

Zunächst wurden eine narrative Literaturrecherche und problemzentrierte Expert*innen-Interviews durchgeführt. Die gewonnenen Daten wurden analysiert und dienten als Grundlage für eine zwei Runden umfassende Delphi-Befragung zur Priorisierung von Forschungsinhalten. Die Ergebnisse bildeten die Basis für die Formulierung der Forschungsagenda.

### Narrative Literaturrecherche

Im Rahmen eines narrativen Literaturreviews wurden Grundlagenwerke der Rehabilitationswissenschaften, relevante Publikationen von universitären, staatlichen und nichtstaatlichen Institutionen sowie internationale wissenschaftliche Publikationen via Google Scholar, Medline via PubMed und CINAHL recherchiert. Die Suchbegriffe waren: Rehabilitation (rehabilitation), Forschungsagenda (research agenda) und Forschungsthemen (research topics), mit einem Fokus auf Publikationen ab 2001 in Englisch oder Deutsch. Die Recherche fand von April bis Juni 2021 statt. Aus den 178 gesichteten Publikationen ergaben sich sieben Themenschwerpunkte: Patient*innenorientierung, Instrumente, spezifische Krankheitsbilder, Herausforderungen/Ressourcen, Wirksamkeit/Nachhaltigkeit, Arbeitskontext und Forschungsinfrastruktur.

### Sampling für Interviews und Delphi-Befragung

Die Stichprobe aus Expert*innen für die mündlichen und schriftlichen Befragungen wurde durch random purposeful sampling 9 10 und Schneeballverfahren gebildet. Ziel war es, einen möglichst breiten Querschnitt unterschiedlicher professioneller Akteur*innen der Rehabilitation mit Expertise aus der Rehabilitationspraxis, -verwaltung und/oder -wissenschaft zu rekrutieren: (1) Jeweils ein*e zufällig ausgewählte*r Praktiker*in aus den Fachdisziplinen Medizin, Pflege, Psychologie, Physiotherapie, Sportwissenschaft, Ergotherapie und Diätologie aus 17 Rehabilitationszentren der PV; (2) alle Mitglieder der ärztlichen, pflegerischen und administrativen Leitung dieser Zentren; (3) Expert*innen der Sozialversicherung in Österreich und Deutschland, darunter Verwaltungs- und Führungskräfte sowie Qualitätsbeauftragte; (4) Wissenschaftler*innen aus Österreich, Deutschland und der Schweiz, die innerhalb der letzten 10 Jahre zumindest eine rehawissenschaftliche Publikation veröffentlicht haben (Zufallsauswahl). Die Teilnahme war freiwillig, vertraulich und datenschutzkonform. Details zur Zusammensetzung der Samples und Rücklaufquoten finden sich im Zusatzmaterial ( **Tab. S1** ).

### Problemzentrierte Interviews

Von April bis November 2021 wurden teilstrukturierte, leitfadengestützte Interviews mit 52 Reha-Expert*innen aus dem DACH-Raum durchgeführt, um individuelle Erkenntnisinteressen, potenzielle Forschungsinhalte, sowie erforderliche Rahmenbedingungen (Forschungsinfrastruktur) und Ressourcen für Forschungskooperationen zu explorieren.

Die Interviews, die im Durchschnitt 61 Minuten (29 bis 144 Minuten) dauerten, wurden transkribiert 11 und mit der Software MaxQDA (Version 2020.4.1) inhaltsanalytisch ausgewertet 12 . Das vorab definierte Kategoriensystem wurde während der Auswertung angepasst und ergänzt. Anhand der Expert*innen-Interviews wurden 79 Inhalte identifiziert, welche die Ergebnisse der Literaturrecherche ergänzten und in die Delphi-Befragung einflossen.

### Delphi-Befragung

Die Delphi-Befragung diente dazu, inhaltlichen Konsens bzw. Dissens unter den Expert*innen zu ermitteln 13 . In zwei Befragungsrunden beurteilten die Expert*innen die Priorität der vorgelegten Forschungsinhalte und ergänzten weitere Inhalte im offenen Antwortformat.

### Durchführung und Erhebung

Die Datenerhebung erfolgte zwischen 19.05.2023 und 20.06.2023 (1. Befragungsrunde) sowie zwischen 04.07.2023 und 01.08.2023 (2. Befragungsrunde) über das Online-Befragungstool SoSci Survey (Version 3.5.0.1).

Der Fragebogen der ersten Befragungsrunde umfasste acht thematische Blöcke mit 102 potenziellen Forschungsinhalten, die in verständlicher Sprache formuliert und bei Bedarf mit kurzen Definitionen bzw. Beispielen ergänzt wurden. Die Inhalte wurden auf einer 5-stufigen Likert-Skala (1=keine bis 5=sehr hohe Priorität) bewertet. Die vorangestellte Frage lautete: «Bitte stufen Sie ein, welche Priorität die folgenden Inhalte/Bereiche in der zukünftigen Rehaforschung haben sollten.». Zudem wurden offene Antworten sowie soziodemografische und tätigkeitsbezogene Daten der Expert*innen eingeholt, die im Zusatzmaterial zu finden sind ( **Tab. S2** ). Die Ergebnisse der ersten Runde wurden zusammengefasst und den Expert*innen in der zweiten Runde anonymisiert rückgemeldet. Hochpriorisierte Forschungsinhalte wurden bereits an dieser Stelle in die Forschungsagenda aufgenommen.

In der zweiten Befragungsrunde wurden 22 Inhalte mit geringer Priorität erneut abgefragt, um vor dem Hintergrund der Gruppenergebnisse eine nochmals reflektierte Einschätzung zu erhalten. Zusätzlich wurden 14 potenzielle Forschungsinhalte, die sich aus den offenen Antworten der ersten Befragungsrunde ergaben und nicht bereits bestehenden Inhalten zugeordnet werden konnten, in der zweiten Runde erstmals bewertet. Die Befragung konnte ohne vollständige Bewertung aller Inhalte abgeschlossen werden, dennoch wurden durchschnittlich rund 93% (Runde 1) bzw. 94% (Runde 2) der Inhalte bewertet.

### Auswertung der Prioritätseinschätzungen

Die Auswertung erfolgte mittels deskriptiver Statistik unter Berechnung von Median, Interquartilsabstand, Mittelwert und Standardabweichung. Als Maß für die Priorisierung wurden die Anteile der Expert*innen herangezogen, die die einzelnen Items mit hoher (Antwort 4 und 5), neutraler (Antwort 3) und niedriger Priorität (Antwort 1 und 2) bewerteten. Ergänzend wurde der Consensus-Wert nach Tastle & Wierman 14 als Kriterium für Konsens bzw. Dissens in der Expert*innengruppe berechnet. Dieser Consensus-Wert liegt zwischen 0 und 1, wobei 0 gar keinen und 1 maximalen Konsens bedeutet. Hoher Konsens (≥0,5) lag vor, wenn mindestens 60% der Expert*innen einem Forschungsinhalt hohe Priorität (Antworten 4 und 5) beigemessen haben. Diese Inhalte wurden bereits nach der 1. Befragungsrunde in die Forschungsagenda aufgenommen. Inhalte, die in keiner Runde von mindestens 20% der Expert*innen als hochprioritär eingestuft wurden, sollten nicht berücksichtigt werden. Tatsächlich unterschritt jedoch keiner der Forschungsinhalte diese Schwelle, sodass alle abgefragten Inhalte letztlich in die Forschungsagenda aufgenommen wurden.

## Ergebnisse

Auf Grundlage der Erkenntnisse aus der Literaturrecherche, den Expert*innen-Interviews und der Delphi-Befragung konnten 116 Forschungsinhalte identifiziert werden. Diese Inhalte wurden in 32 Forschungsthemen, elf Forschungsfelder und vier Forschungsbereiche mit zunehmendem Abstraktionsgrad geordnet.

**Abb. 1:**
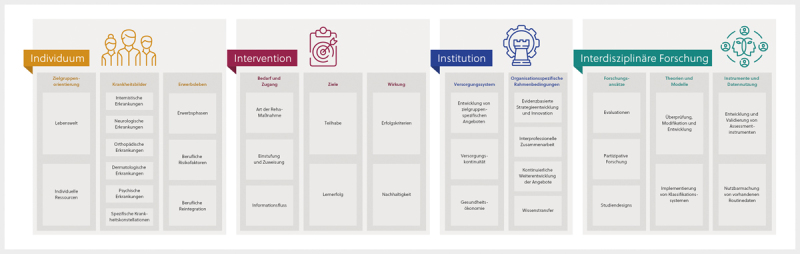
Überblick der Forschungsagenda.

Mit den vier übergeordneten Forschungsbereichen Individuum, Intervention, Institution und Interdisziplinäre Forschung werden unterschiedliche Betrachtungsebenen der Gegenstandsbereiche und methodisch-theoretischen Grundlagen der Rehaforschung abgebildet ( Abb. 1 ). Die Bereiche sind damit grundsätzlich voneinander abgrenzbar, stehen aber in enger Beziehung und Wechselwirkung zueinander.

Im Folgenden werden die vier Bereiche einleitend definiert, bevor eine Untergliederung und Präzisierung anhand zugehöriger Forschungsfelder (im Ergebnisteil unterstrichen) und Forschungsthemen (im Ergebnisteil *kursiv* ) erfolgt. Für jeden der vier Forschungsbereiche werden jeweils fünf konkrete, auf diese Weise aggregierte und besonders konsensfähige Forschungsinhalte wiedergegeben. Alle bewerteten Inhalte werden sprachlich gekürzt als Zusatzmaterial zur Verfügung gestellt ( **Tab. S3-S6** ).

### Forschungsbereich Individuum

Die Hauptaufgaben der Rehabilitation bestehen darin, gesundheitsbedingte Einschränkungen von Rehabilitand*innen zu beseitigen oder weitest möglich zu reduzieren. Die Betroffenen sollen befähigt werden, mit eventuell verbleibenden gesundheitlichen Beeinträchtigungen kompetent und selbstbestimmt umzugehen. Sie sollen nachhaltig in die Lage versetzt werden, ihre Funktionen in Gesellschaft, Familie und Beruf wahrzunehmen und damit aktiv an allen relevanten Lebensbereichen teilzuhaben. Um die Zielgruppenorientierung der Rehabilitationsmaßnahmen zu gewährleisten ist die Orientierung am Individuum unabdingbar. Für die Rehabilitationspraxis und -forschung müssen sowohl die gesundheitlichen Defizite, als auch die *individuellen Ressourcen* und die *Lebenswelten* der Rehabilitand*innen richtungsweisend sein.

Die Krankheitsbilder ( *internistische, neurologische, orthopädische, dermatologische, psychische Erkrankungen und spezifische Krankheitskonstellationen* ) stehen nicht per se im Fokus der Rehaforschung, sondern die damit verbundenen indikationsspezifischen Problemlagen und Handlungsmöglichkeiten der Betroffenen.

Das Erwerbsleben als wichtige Teilhabemöglichkeit steht im Fokus der Rehaforschung. Zentrale Forschungsthemen sind daher *berufsbedingte Risikofaktoren* für ein vorzeitiges berufliches Ausscheiden, Herausforderungen in unterschiedlichen Lebens- und *Erwerbsphasen* sowie Wege, um Arbeitsfähigkeit zu erhalten und *berufliche Reintegration* nach der Rehabilitation zu fördern.

Im Forschungsbereich Individuum wurden erwerbsfähige Personen mit hoher Wahrscheinlichkeit einer beruflichen Wiedereingliederung (91,6%), Motivation zur Mitwirkung an Rehabilitationsmaßnahmen (89,4%), psychische Arbeitsbelastung (89,0%), Frühinterventionen im Erwerbsprozess (87,7%) und die Gestaltung der Nahtstelle zwischen Rehabilitation und beruflicher Reintegration (87,6%) als besonders prioritäre Forschungsinhalte bewertet.

### Forschungsbereich Intervention

Interventionen in der Rehabilitation umfassen alle Schritte, die von verschiedenen Gesundheitsprofessionen gesetzt werden, um gemeinsam mit Rehabilitand*innen individuelle Rehabilitationsziele zu erreichen. Es handelt sich dabei um bedarfsorientierte, evidenzbasierte, professionelle oder professionell initiierte Aktivitäten und Maßnahmen mit präventiver, therapeutischer oder gesundheitsfördernder Zielsetzung.

Um sicherzustellen, dass Rehabilitationsmaßnahmen zielgerichtet wirken können, ist der Bedarf und Zugang zu entsprechenden Interventionen ein zentrales Forschungsfeld. Komplexe, individuelle Problemfelder der Rehabilitand*innen erfordern unterschiedliche *Arten von Rehabilitationsmaßnahmen,* die medizinisch, beruflich oder medizinisch-beruflich ausgerichtet sein können und in ambulanten, stationären oder telemedizinischen Settings durchgeführt werden. Damit verbunden sind Fragen der adäquaten *Einstufung* und Bewertung des individuell notwendigen und zumutbaren Rehabilitationsumfangs, der Möglichkeiten der Digitalisierung in der Rehabilitationsplanung sowie der passgenauen und zeitnahen *Zuweisung* von Rehabilitand*innen zu konkreten Rehaprogrammen. Von grundlegender Bedeutung ist daher die Betrachtung der *Informationsflüsse* von den Rehabilitationsträgern in die (Fach-)Öffentlichkeit, z. B. die Frage, wie und in welchem Umfang Patient*innen (=potenzielle Rehabilitand*innen) und Zuweiser*innen die notwendigen Informationen über die Angebote und Potenziale der Rehabilitation sowie über die erforderliche Antragstellung für diese Leistungen erhalten.

Die konkreten Ziele, die mit den Interventionen in verschiedenen Zeiträumen angestrebt werden, basieren auf einem bio-psycho-sozialen Gesundheitsverständnis und orientieren sich an den individuellen Ressourcen des Einzelnen. Die Ziele sind naturgemäß sehr individuell und können im Verlauf der Rehabilitation variieren. Das Hauptziel aller Rehabilitationsmaßnahmen ist die Wiederherstellung oder Verbesserung der beruflichen und sozialen *Teilhabe* der Rehabilitand*innen. Um dieses Ziel mittelfristig zu erreichen und die (wieder-)hergestellten Teilhabemöglichkeiten längerfristig zu sichern, ist der *Lernerfolg* der Rehabilitand*innen ein zentrales Schlüsselelement. Der Lernerfolg bezieht sich im engeren Sinne auf den Zuwachs an gesundheitsbezogenem und krankheitsspezifischem Wissen, welches den Rehabilitand*innen in Schulungen vermittelt wird. Im weiteren Sinne umfasst der Lernerfolg auch die langfristige, selbstständige und im Alltag integrierte Anwendung gesundheitsbezogener Kompetenzen und Fähigkeiten.

Die Wirkung von Rehabilitation ist sowohl in der Rehapraxis als auch in der Rehaforschung von fundamentaler Bedeutung. Die konkreten Wirkungen und Erfolge zielgruppenspezifischer Interventionen können nur anhand ebenso zielgruppenspezifischer *Erfolgskriterien* bzw. Outcomes adäquat identifiziert und gemessen werden. Neben problemorientierten Kriterien und Assessments, die objektive Krankheitsindikatoren bis hin zur subjektiven Symptombelastung abbilden, sind zahlreiche bio-psycho-soziale Faktoren und Mechanismen zu identifizieren, adäquat zu operationalisieren und in der Forschung zu berücksichtigen, um (ausbleibende) Rehabilitationserfolge erklären und vorhersagen zu können. Die *Nachhaltigkeit* der im Rahmen der Rehabilitation erzielten Effekte bzw. Erfolge stellt aus individueller und gesundheitsökonomischer Sicht einen dringenden und zunehmend prioritären Forschungsinhalt dar. Im Mittelpunkt stehen nachhaltige Veränderungen und Entwicklungen in der beruflichen und sozialen Teilhabe, aber auch des dafür grundlegenden Gesundheitszustandes, der Lebensqualität und der Selbstständigkeit der Rehabilitand*innen.

Im Forschungsbereich Intervention wurden die passgenaue Zuweisung zu Rehabilitationsprogrammen (92,0%), die Nachhaltigkeit von Interventionen hinsichtlich der beruflichen Teilhabe (91,3%), medizinische Rehabilitationsmaßnahmen (90,7%), berufliche Teilhabe von Rehabilitand*innen als Interventionsziel (88,7%) und die Nachhaltigkeit von Interventionen auf den Gesundheitszustand (86,6%) als besonders prioritäre Forschungsinhalte bewertet.

### Forschungsbereich Institution

Rehabilitation ist eine komplexe Leistung, die in die Strukturen des Gesundheits- und Sozialversicherungssystems eingebettet ist. Rehaforschung befasst sich mit inhärent systemischen Gegenstandsbereichen, die in ein umfassendes Versorgungssystem eingebettet sind. Dieser Umstand muss bei der Gestaltung und *Entwicklung von Rehabilitationsangeboten* berücksichtigt werden, die spezifische Zielgruppen und Gesundheitsziele erreichen sowie systemische Versorgungsfunktionen und *gesundheitsökonomische* Kriterien erfüllen sollen. Die notwendige und bestmögliche Gewährleistung von *Versorgungskontinuität* erfordert eine sorgfältige Betrachtung der Behandlungspfade im Rehabilitationssystem sowie der Schnittstellen und Pfade der Rehabilitand*innen im Sozialversicherungs- und Gesundheitssystem vor und nach der Rehabilitation.

Die Gestaltung und Umsetzung der Rehabilitation in Österreich wird von organisationsspezifischen Rahmenbedingungen geprägt, welche Kontextbedingungen und Impulse für die Rehaforschung darstellen, aber auch die *evidenzbasierte Strategieentwicklung und Innovation* in der Rehabilitation mitbestimmen. Organisationsspezifische Rahmenbedingungen umfassen interne Grundhaltungen, professionelle Leitbilder und strategische Zielüberlegungen, die wesentlich zu qualitativ hochwertiger und effektiver Rehabilitation beitragen. Konkrete organisationsspezifische Forschungsinhalte finden sich in nahezu allen Bereichen der Rehabilitation und in der organisationsinternen Kommunikation und Nutzung der generierten wissenschaftlichen Evidenz. Die *kontinuierliche Weiterentwicklung von Rehabilitationsangeboten* , Therapiestandards und digital- bzw. technikgestützten Rehabilitationsmaßnahmen sind sowohl Grundhaltungen der Organisation als auch konkrete Forschungsinhalte. Gleiches gilt für den aktiven *Wissenstransfer* wissenschaftlicher Erkenntnisse in die Rehapraxis und die *interprofessionelle Zusammenarbeit* im Reha-Team.

Der Zusammenarbeit im interdisziplinären Reha-Team sowie die Entwicklung von Therapiestandards sind für die Befragten hochprioritäre Forschungsinhalte (jeweils 84,5%). Dem Transfer wissenschaftlicher Erkenntnisse in die Praxis und der Entwicklung von Angeboten zur Sekundärprävention wird gleichhohe Priorität (jeweils 83,8%) eingeräumt, gefolgt von der Entwicklung von Angeboten zur Tertiärprävention (81,8%).

### Forschungsbereich Interdisziplinäre Forschung

Praktiker*innen in der Rehabilitation begegnen einer Vielzahl von Rehabilitand*innen mit komplexen und individuellen gesundheitlichen Problemlagen, die von Angehörigen einer einzelnen Gesundheitsprofession weder quantitativ noch qualitativ vollständig und adäquat adressiert werden können. Verschiedene fachspezifische Kompetenzen müssen in interdisziplinären Rehabilitationsteams im Sinne der Rehabilitand*innen kombiniert und abgestimmt werden. Gleichermaßen ist Rehaforschung unweigerlich interdisziplinär durchzuführen, um qualitativ hochwertige, zuverlässige und für das gesamte Feld der Rehabilitation relevante Ergebnisse zu generieren.

Rehaforschung ist die systematische Suche nach validem, anwendungsorientiertem und praxisrelevantem Wissen. Ein Pluralismus der Forschungsansätze ist unabdingbar, um den Anforderungen in der Rehaforschung gerecht zu werden und Forschung zu betreiben, die der inhaltlichen Vielfalt sowie dem aktuellen Stand der Evidenz und Theorieentwicklung in den Rehawissenschaften angemessen ist. Hierzu zählen insbesondere auch Ansätze der *partizipativen Forschung* und der *Evaluation* bestehender als auch neuer Strukturen und Programme in der Rehabilitation.

Das letztendlich von den Forscher*innen gewählte *Studiendesign* ist abhängig von den Fragestellungen und den Erkenntnisinteressen, dem Umfang und Reifegrad des diesbezüglichen rehawissenschaftlichen Wissensbestandes sowie den vorgegebenen Rahmenbedingungen in der anwendungsorientierten Feldforschung. Die generierten Ergebnisse müssen umfassend abgesichert, verlässlich und damit therapeutisch relevant sein. Erkenntnisse auf diesem hohen Evidenzniveau erfordern einen homogenen Bestand an qualitativ hochwertigen experimentellen Primärstudien oder entsprechende Studienreihen. Diese Studien wiederum setzen voraus, dass zuvor relevante und aussagekräftige nicht-experimentelle Forschung durchgeführt wird. Dazu gehören Studien zur Exploration spezifischer Phänomene in bestimmten Populationen (explorative Studien), umfassende und detaillierte Beschreibungen beobachtbarer oder messbarer Sachverhalte unter natürlichen Bedingungen (deskriptive Studien) sowie quasi-experimentelle Studien, die unter den unterschiedlichen Rahmenbedingungen der Rehapraxis durchgeführt werden. Der Forschungsbedarf in der vergleichsweise jungen und vor allem in Österreich noch im Entstehen begriffenen Disziplin der Rehabilitationswissenschaften ist evident.

Das Potenzial der Rehaforschung kann nicht in einem theoriefreien Raum ausgeschöpft werden. Theorien und Modelle sind unverzichtbare Ergebnisse, Interpretationsrahmen und Ausgangspunkte für empirische Erkenntnisse. Sie fördern das Verständnis der Wissenschaft für die Praxis, können kontinuierlich überprüft, erweitert oder verworfen werden und verdeutlichen, welche impliziten und expliziten Annahmen und Wirkmechanismen professionellem Handeln zugrunde liegen („Programmtheorien“). Theoretische und modellhafte Grundlagen spiegeln sich in der Rehapraxis auch in unterschiedlichen *Klassifikationssystemen* wider, die in multiprofessionellen Teams eingesetzt werden. Insbesondere die Internationale Klassifikation der Funktionsfähigkeit, Behinderung und Gesundheit (ICF) der Weltgesundheitsorganisation hat sich in der Rehabilitation etabliert, da sie eine gemeinsame interdisziplinäre Sprache sowie ein ganzheitliches und teilhabeorientiertes therapeutisches Verständnis fördert.

Die angemessene und präzise Erfassung individueller Problem-, Bedürfnis- und Ressourcenkonstellationen in der Praxis und Forschung steht im Zentrum des Feldes Instrumente und Datennutzung. Aufgabe der Rehaforschung ist es, *Assessmentinstrumente* zu entwickeln, zu validieren und für den spezifischen Kontext der Rehabilitation zu adaptieren. Darüber hinaus sollten in der Rehabilitation verfügbare *Routinedaten* und Registerdaten erschlossen und genutzt werden, um zukunftsorientierte Planungen im österreichischen Rehabilitationssystem zu unterstützen.

Im Bereich der Interdisziplinären Forschung wurde partizipative Forschung gemeinsam mit Praktiker*innen (78,5%) und ebenso mit Rehabilitand*innen (75,0%) von der Expert*innengruppe hoch priorisiert. Es bestand ein hoher Konsens bezüglich der Priorität der Nutzbarmachung bereits vorhandener personenbezogener Routinedaten (75,3%), der wissenschaftlichen Begleitung neuer Maßnahmen (75,0%) und der Evaluationen bestehender Heilverfahren (69,3%).

## Diskussion

Die vorliegende Forschungsagenda für Rehaforschung ist ein wichtiger Schritt zur systematischen Strukturierung und (Weiter-)Entwicklung der Forschungsaktivitäten in der österreichischen PV. Die multimethodische und multiperspektivische Entwicklung erlaubte es einen breiten Überblick über aktuell relevante Forschungsthemen zu generieren und Forschungsprioritäten aus Expert*innensicht zu explizieren.

Die Identifikation von 116 Inhalten, 32 Themen und elf Feldern in den vier Forschungsbereichen Individuum, Intervention, Institution und Interdisziplinäre Forschung spiegelt die Vielfalt, Vielschichtigkeit und Komplexität der potentiellen Gegenstandsbereiche der Rehaforschung in der PV wider. Die hohe Forschungspriorität, die den vielfältigen Forschungsinhalten aller vier Bereiche von den Expert*innen beigemessen wurde, kann auch als Sinnbild für den momentanen Status quo in der Rehaforschung und -praxis verstanden werden: Die inhaltlichen Schwerpunkte spiegeln den aktuellen Evidenzstand in der Rehaforschung und deren Lücken ebenso wider wie die professionsübergreifenden Herausforderungen und Ressourcen in allen Bereichen der Rehabilitation. All dies sind zugleich direkte Appelle an Reha-Forscher*innen, entsprechende Forschung inhaltlich und methodisch voranzutreiben, um den komplexen Herausforderungen in der Reha gerecht zu werden.

Der Bereich Individuum fokussiert auf die Bedürfnisse, Herausforderungen und Ressourcen von Rehabilitand*innen und umfasst die Forschungsfelder *Zielgruppenorientierung* , *Krankheitsbilder* und *Erwerbsleben* . Das Individuum als zentralen Akteur in der Rehabilitation und in seiner Ganzheitlichkeit zu verstehen, eröffnet Möglichkeiten zur Erforschung und Entwicklung bedarfsgerechter Rehabilitationsmaßnahmen.

Im Bereich Intervention stehen die Forschungsfelder *Bedarf und Zugang* , *Ziele* sowie *Wirkung* im Mittelpunkt. Die Forschung widmet sich vor allem der Identifikation von Zugangsbarrieren zur Rehabilitation, der zielgerichteten Gestaltung und Durchführung von Rehabilitation sowie der Förderung des Verständnisses, der Nachweisbarkeit und der Nachhaltigkeit ihrer Wirkungen.

Der Forschungsbereich Institution umfasst mit den Forschungsfeldern *Versorgungssystem* und *Organisationsspezifische Rahmenbedingungen* die Strukturen, Regeln und Bedingungen, die das (gesundheits-)systemische Fundament der Rehabilitationspraxis bilden. Rehabilitation wird hier als eine durch organisationsinterne Strukturen geprägte und in das umfassendere Gesundheits- und Sozialsystem eingebettete Leistung verstanden und erforscht.

Die Grundlagen und Instrumente der inhärent interdisziplinären Rehaforschung werden im Bereich Interdisziplinäre Forschung herausgearbeitet und in den Forschungsfeldern *Designs und Methoden* , *Theorien und Modelle* sowie *Instrumente und Datennutzung* abgebildet. Diese Forschungsgrundlagen müssen sorgfältig geprüft, angepasst und weiterentwickelt werden, um qualitativ hochwertige und aussagekräftige Forschung durchführen zu können, die wesentlich zur evidenzbasierten Gestaltung und Weiterentwicklung der Rehabilitationspraxis beitragen kann.

### Reflexion der Ergebnisse und Methodik

Die vorliegende Forschungsagenda reiht sich in den Kanon bestehender Forschungsagenden im Bereich der Rehabilitation ein. In Übereinstimmung mit den Erkenntnissen von Bickenbach & Danermark 15 wird auch hier die zentrale Bedeutung einer interdisziplinären Perspektive in der Rehaforschung betont. Ebenso entspricht die hohe Priorität partizipativer Forschungsansätze den Empfehlungen von Schaller et al. 16 . Eine Besonderheit stellt hingegen die schrittweise Abstraktion der Forschungsinhalte dar, wie sie in dieser Forschungsagenda vorgenommen wurde. Durch dieses Vorgehen wurde nicht nur ein Orientierungssystem geschaffen, sondern auch der Blick für Interdependenzen und Wechselwirkungen bei der Planung, Umsetzung und Erforschung einer effektiven und personenzentrierten Rehabilitation geschärft.

Um die vielfältigen Gegenstandsbereiche der Rehaforschung im Kontext der PV möglichst umfassend abzubilden, wurde bewusst auf die Formulierung spezifischer Forschungsfragen verzichtet. Auf diese Weise kann auch flexibler und dynamischer auf sich verändernde Anforderungen und neue Entwicklungen in der Rehabilitation reagiert werden. Konkrete Fragestellungen können entsprechend den jeweiligen Erkenntnisinteressen entwickelt und dennoch eindeutig in der Forschungsagenda verortet werden.

Auch der gewählte multimethodische Entwicklungsansatz stellt einen Unterschied zu anderen Forschungsagenden dar, die etwa primär auf Basis einer Literaturrecherche 17 oder ausschließlich auf Basis einer Delphi-Befragung 18 entwickelt wurden. Gerade in der Kombination von Literatur, Expert*innen-Interviews und Delphi-Befragung zeigte sich jedoch ein großes Potential für eine sorgfältige, umfassende und tiefgehende Annäherung an die facettenreichen Erkenntnisinteressen in der Rehabilitation. Als Indiz dafür kann gewertet werden, dass keiner der in die Delphi-Befragung einbezogenen Inhalte aufgrund mangelnden Konsenses nicht in die Forschungsagenda aufgenommen wurde, wobei das Kriterium für die Nichtaufnahme (Konsens<20%) bewusst wenig restriktiv gewählt wurde. Dies unterstreicht letztlich, dass die bewerteten Forschungsinhalte von interprofessioneller bzw. interdisziplinärer Relevanz sind und zur Weiterentwicklung der Rehabilitation insgesamt beitragen können. Nicht nur der wechselseitige Wissenstransfer, sondern auch die kontinuierliche Zusammenarbeit zwischen Forschung und Praxis wird dabei ein Schlüsselelement sein, um das Feld der Rehabilitation nachhaltig positiv zu beeinflussen.

### Limitationen

Die Forschungsagenda wurde spezifisch für die österreichische Pensionsversicherung entwickelt und ist daher nicht unmittelbar auf andere Kontexte übertragbar, deren Spezifika bei der Entwicklung nicht berücksichtigt wurden. Hervorzuheben ist auch, dass die Rehabilitand*innen nicht direkt in den Entwicklungsprozess einbezogen wurden, um ihre Interessen und Anliegen noch deutlicher zum Ausdruck zu bringen. Im Rahmen der Entwicklung von Forschungsagenden, die beispielsweise spezifische Versorgungssituationen fokussieren, ist eine verstärkte Partizipation der Rehabilitand*innen zu empfehlen.

### Fazit

Die multimethodische und multiperspektivische Entwicklung der Forschungsagenda ermöglichte eine umfassende Annäherung an die komplexe Themenvielfalt in der Rehabilitation. Die Forschungsagenda ist spezifisch für die österreichische Pensionsversicherung entwickelt worden, ihre zentralen Erkenntnisse werden aber auch andernorts als Orientierungshilfe, struktureller Rahmen und Diskussionsgrundlage in der zukünftigen Rehaforschung dienlich sein. Unmittelbar gilt es den diesbezüglichen Diskurs mit den Vertragspartnereinrichtungen der PV, anderen Sozialversicherungsträgern sowie akademischen Forschungs- und Bildungseinrichtungen zu intensivieren. Interdisziplinäre, partizipative und theoriegeleitete Forschungsansätze werden jedenfalls entscheidende Impulse liefern, um die Rehabilitation effektiv zu gestalten und kontinuierlich weiterzuentwickeln.

## Kernbotschaft

Eine multiperspektivisch entwickelte Forschungsagenda ist entscheidend, um sicherzustellen, dass sich Rehaforschung möglichst stark an den konkreten, aktuellen und mitunter divergierenden Erkenntnisinteressen der Praktiker*innen, Wissenschaftler*innen und Verwaltungskräfte im Bereich der Rehabilitation orientiert. Sie eröffnet und strukturiert breite interdisziplinäre und anwendungsorientierte Forschungsfelder, die auf der Mikro-, Meso- und Makroebene untersucht werden können.

## Zusätzliches Material

Zusätzliches Material Zusätzliches Material
