# Supplementary material for: Development of an agenda for rehabilitation research in the Austrian pension insurance system
Source: Rehabilitation (Stuttg). 2025 Jun 10;64(3):139–45. [Article in German] doi: 10.1055/a-2563-6776 (PMC12173632; doi:10.1055/a-2563-6776)
Supplement: Supplementary file 1 — Zusätzliches Material [file 10-1055-a-2563-6776-2024-04-0017.pdf]

Tabelle S1: Tätigkeitsbereich der Teilnehmenden: Interviews und Delphi-Befragung samt Rücklauf

	Interviews	Delphi-Befragung*				
			Runde 1		Runde 2	
**Tätigkeitsbereich**	**Anzahl**	**eingeladen**	**Teiln.**	**Rücklauf**	**Teiln.**	**Rücklauf**
Wissenschaft	18	230	53	23,0 %	42	18,3 %
Wissenschafter*innen Österreich	8	100	24	24,0 %	15	15,0 %
Wissenschaftler*innen Deutschland	5	100	24	24,0 %	22	22,0 %
Wissenschafter*innen Schweiz	5	30	5	16,7 %	5	16,7 %
Mitglieder der Verwaltung der Sozialversicherung	16	41	32	78,0 %	23	56,1 %
Mitglieder der Verwaltung	14	24	21	87,5 %	12	50,0 %
Qualitätsbeauftragte der PV	2	17	11	64,7 %	10	58,8 %
Mitglieder der KOFÜ der Eigenen Einrichtungen der PV	11	49	36	73,5 %	33	67,3 %
Ärztliche Leitung	6	17	16	94,1 %	14	82,4 %
Pflegedienstleitung	4	15	8	53,3 %	10	66,7 %
Verwaltungsleitung	1	17	12	70,6 %	9	52,9 %
Praktiker*innen in den Eigenen Einrichtungen der PV	7	119	42	35,3 %	54	45,4 %
Mediziner*innen	-	17	5	29,4 %	3	17,6 %
Pflegekräfte	1	17	3	17,6 %	6	35,3 %
Psycholog*innen	1	17	10	58,8 %	7	41,2 %
Physiotherapeut*innen	2	17	5	29,4 %	7	41,2 %
Sportwissenschaftler*innen	1	17	10	58,8 %	12	70,6 %
Ergotherapeut*innen	-	17	3	17,6 %	10	58,8 %
Diätolog*innen	2	17	6	35,3%	9	52,9%
Gesamt	52	439	163	37,1%	151	34,4%

*In beiden Runden wurden durchschnittlich über 90 % der abgefragten Inhalte bewertet (Runde 1: 93 %, Runde 2: 94 %).

Tabelle S2: Demografische Daten der Teilnehmenden der Delphi-Befragung

		Runde 1		Runde 2	
		Anz.	%	Anz.	%
**Geschlecht**	Frauen	64	46,0 %	77	58,8 %
	Männer	75	54,0 %	54	41,2 %
	Gesamt gültig	139	100 %	131	100 %
	Fehlende	24	14,7 %	21	13,8 %
**Alter**	bis 30 Jahre	8	5,6 %	11	7,9 %
	31-40 Jahre	20	14,1 %	26	18,7 %
	41-50 Jahre	49	34,5 %	42	30,2 %
	51 Jahre und älter	65	45,8 %	60	43,2 %
	Gesamt gültig	142	100 %	139	100 %
	Fehlende	21	12,9 %	13	8,6 %
**Alter nach Geschlecht**	Frauen bis 30 Jahre	7	5,1 %	10	7,6 %
	Männer bis 30 Jahre	1	0,7 %	1	0,8 %
	Frauen 31-40 Jahre	10	7,2 %	14	10,7 %
	Männer 31-40 Jahre	10	7,2 %	10	7,6 %
	Frauen 41-50 Jahre	26	18,8 %	25	19,1 %
	Männer 41-50 Jahre	21	15,2 %	15	11,5 %
	Frauen 51 Jahre und älter	21	15,2 %	28	21,4 %
	Männer 51 Jahre und älter	42	30,4 %	28	21,4 %
	Gesamt gültig	138	100 %	131	100 %
	Fehlende	25	15,3 %	21	13,8 %
**Erfahrung im Reha-Bereich**	unter 1 Jahr	8	5,8 %	5	3,6 %
	1-2 Jahre	7	5,1 %	6	4,3 %
	3-5 Jahre	24	17,4 %	28	20,1 %
	6-10 Jahre	26	18,8 %	27	19,4 %
	mehr als 10 Jahre	73	52,9 %	73	52,5 %
	Gesamt gültig	138	100 %	139	100 %
	Fehlende	25	15,3 %	13	8,6 %
**Führungsposition**	Führungsposition	87	60,8 %	76	53,5 %
	keine Führungsposition	56	39,2 %	66	46,5 %
	Gesamt gültig	143	100 %	142	100 %
	Fehlende	20	12,3 %	10	6,6 %

Tabelle S3: Prioritätseinschätzungen im Forschungsbereich Individuum

FORSCHUNGSBEREICH INDIVIDUUM	n	MW	SD	Priorität hoch in %	Priorität mittel in %	Priorität niedrig in %
**Zielgruppenorientierung**						
Lebenswelt						
Einbeziehen des privaten/familiären Umfeldes	159	4,04	0,99	**73,0 %**	20,7 %	6,3 %
altersspezifische Fragestellungen	159	3,86	1,02	**66,7 %**	23,9 %	9,4 %
Personen mit Betreuungspflichten	142	3,80	1,13	**62,7 %**	26,1 %	11,3 %
Fragestellungen zum sozioökonomischen Hintergrund	149	3,26	1,12	**45,0 %**	32,1 %	18,9 %
Fragestellungen zum Migrationshintergrund	149	2,99	1,22	**35,6 %**	26,8 %	37,6 %
genderspezifische Fragestellungen	149	2,85	1,30	**32,9 %**	27,5 %	39,6 %
Individuelle Ressourcen						
Motivation zur Mitwirkung an der Reha-Maßnahme	160	4,50	0,85	**89,4 %**	6,2 %	4,4 %
Eigenverantwortung der Rehabilitand*innen	160	4,44	0,84	**87,5 %**	7,5 %	5,0 %
Gesundheitskompetenz der Rehabilitand*innen	160	4,24	0,86	**84,4 %**	10,6 %	5,0 %
individuelle Krankheitsbewältigung	160	4,18	0,78	**83,8 %**	13,7 %	2,5 %
zielgruppenspezifische Hilfsmittelversorgung	143	3,85	1,13	**67,1 %**	18,9 %	14,0 %
**Krankheitsbilder**						
Dermatologische Erkrankungen						
dermatologische Erkrankungen	143	3,10	1,12	**35,7 %**	35,0 %	29,4 %
Internistische Erkrankungen						
onkologische Erkrankungen	154	4,09	0,84	**75,3 %**	21,4 %	3,2 %
Adipositas	153	4,00	1,02	**72,5 %**	18,3 %	9,1 %
pneumologische Erkrankungen	153	3,97	0,85	**69,9 %**	26,1 %	3,9 %
kardiologische Erkrankungen	154	4,04	0,91	**69,5 %**	27,3 %	3,2 %
Diabetes mellitus	153	3,93	0,87	**68,0 %**	27,4 %	4,6 %
rheumatologische Erkrankungen	142	3,66	1,02	**60,6 %**	27,5 %	12,0 %
gastroenterologische Erkrankungen	142	3,58	1,03	**53,5 %**	31,7 %	14,8 %
nephro-urologische Erkrankungen	142	3,30	1,02	**40,8 %**	38,7 %	20,4 %
Neurologische Erkrankungen						
Schlaganfall	155	4,17	0,85	**76,1 %**	21,3 %	2,6 %
(Poly-) Neuropathien	143	3,84	0,92	**68,5 %**	24,5 %	7,0 %
Parkinson	143	3,79	0,93	**64,3 %**	28,7 %	7,0 %
multiple Sklerose	152	3,79	0,93	**63,8 %**	28,3 %	7,9 %
intellektuelle Behinderung	143	3,29	1,15	**44,1 %**	28,7 %	27,3 %
Krampfleiden	142	3,25	1,14	**43,0 %**	43,0 %	30,3 %
ALS (amyotrophe Lateralsklerose)	143	3,24	1,04	**39,2 %**	38,5 %	22,4 %
Orthopädische Erkrankungen						
orthopädische Erkrankungen	143	4,02	1,02	**76,9 %**	13,3 %	9,8 %
Psychische Erkrankungen						
psychische Beeinträchtigungen	154	4,26	0,95	**77,9 %**	17,5 %	4,5 %
psychosomatische Erkrankungen	153	4,16	1,00	**77,1 %**	15,7 %	7,2 %
psychiatrische Erkrankungen	153	3,99	0,98	**71,9 %**	20,3 %	7,8 %

Forschungsbereich Individuum	n	MW	SD	Priorität hoch in %	Priorität mittel in %	Priorität niedrig in %
Spezifische Krankheitskonstellationen						
chronische Schmerzen	154	4,2	0,92	**79,2 %**	16,2 %	4,5 %
Long-Covid-Erkrankungen	153	3,73	1,16	**65,4 %**	17,6 %	17,0 %
Multimorbidität	155	3,79	1,14	**62,6 %**	23,2 %	14,2 %
erfolgte Organtransplantation	142	3,49	1,08	**47,2 %**	36,6 %	16,2 %
Allergien	143	3,27	1,22	**42,0 %**	30,8 %	27,3 %
**Erwerbsleben**						
Erwerbsphasen						
Personen im erwerbsfähigen Alter, vorrausichtlich wieder eingliederbar	154	4,51	0,67	**91,6 %**	7,8 %	0,6 %
Personen im Erwerbsprozess ("early intervention")	154	4,48	0,74	**87,7 %**	11,0 %	1,3 %
Personen im Jugendalter	154	4,26	1,00	**79,9 %**	10,4 %	9,7 %
Personen im Pensionsalter	154	3,62	1,16	**61,7 %**	20,8 %	17,5 %
Personen im erwerbsfähigen Alter, vorrausichtlich nicht mehr eingliederbar	143	3,51	1,24	**53,8 %**	25,2 %	21,0 %
Berufliche Risikofaktoren						
psychische Arbeitsbelastung	154	4,42	0,91	**89,0 %**	7,1 %	3,9 %
Personen mit besonderen beruflichen Problemlagen	142	4,10	0,96	**78,2 %**	14,1 %	7,7 %
körperliche Arbeitsbelastung	153	3,93	1,01	**68,0 %**	24,8 %	7,2 %
gesundheitsbelastende Arbeitsbedingungen	153	3,86	1,09	**65,4 %**	24,2 %	10,5 %
gesundheitsbelastende Arbeitsumgebung	142	3,7	1,14	**57,0 %**	27,5 %	15,5 %
Berufliche Reintegration						
Nahtstellenmanagement zwischen Reha und Wiedereinstieg	153	4,48	0,80	**87,6 %**	9,8 %	2,6 %
Arbeitsfähigkeit der Rehabilitand*innen	153	4,41	0,85	**86,9 %**	9,8 %	3,3 %
Beitrag der Arbeitgeber*innen zur beruflichen Reintegration	153	3,93	1,01	**68,0 %**	24,8 %	7,2 %
Adaption von Arbeitsplätzen bei gesundheitlichen Einschränkungen	153	3,86	1,09	**65,4 %**	24,2 %	10,5 %

Legende: n= Anzahl; MW= Mittelwert; SD= Standardabweichung (durchschnittliche Streuung um den Mittelwert);
Priorität hoch = Antwort 4 + 5; Priorität mittel = Antwort 3; Priorität niedrig = Antwort 1+2

Tabelle S4: Prioritätseinschätzungen im Forschungsbereich Intervention

FORSCHUNGSBEREICH INTERVENTION	n	MW	SD	Priorität hoch in %	Priorität mittel in %	Priorität niedrig in %
Bedarf und Zugang						
Art der Reha-Maßnahme						
medizinische Reha-Maßnahmen	161	4,50	0,71	**90,7 %**	8,7 %	0,6 %
berufliche Reha-Maßnahmen	161	4,14	0,93	**80,1 %**	14,3 %	5,6 %
soziale Reha-Maßnahmen	150	3,17	1,13	**39,3 %**	31,3 %	29,3 %
Einstufung und Zuweisung						
passgenaue Zuweisung zum "richtigen" Reha-Programm	162	4,54	0,74	**92,0 %**	5,6 %	2,5 %
Erkennen von Früh-Reha-Bedarf	162	4,37	0,89	**83,3 %**	13,0 %	3,7 %
Ermittlung besonderer beruflicher Problemlagen	161	4,26	0,93	**82,0 %**	12,4 %	5,6 %
Einstufung des Reha-Bedarfs ("Reha-Dosis")	162	4,13	0,94	**77,2 %**	17,9 %	4,9 %
Ermittlung der Funktionsfähigkeit der Patient*innen (Aufnahme-Assessment)	150	4,08	1,03	**76,0 %**	14,7 %	9,3 %
Potentiale der Digitalisierung für die Reha-Planung	148	3,99	1,01	**68,2 %**	23,0 %	8,8 %
telemed. Assessment zur Einstufung d. Reha-Bedarfs	142	3,68	1,21	**58,5 %**	26,1 %	15,5 %
Informationsfluss						
Informationszugang bzgl. Reha/Antragstellung für Zuweiser*innen	163	4,09	1,06	**75,5 %**	11,7 %	12,9 %
Informationszugang bzgl. Reha/Antragstellung für Patient*innen	163	3,93	1,10	**65,6 %**	22,1 %	12,3 %
Ziele						
Teilhabe						
berufliche Teilhabe der Rehabilitand*innen	159	4,45	0,87	**88,7 %**	8,2 %	3,1 %
gesellschaftliche und soziale Teilhabe der Rehabilitand*innen	159	4,3	0,92	**82,4 %**	13,2 %	4,4 %
Lernerfolg						
Langfristige Umsetzung des Gelernten in den Alltag	149	4,28	0,83	**81,9 %**	16,1 %	2,0 %
Lernerfolg nach Rehabilitand*innen-Schulungen	143	3,78	1,15	**67,8 %**	16,8 %	15,4 %
Wirkung						
Erfolgskriterien						
Ermittlung relevanter Einflussfaktoren auf Reha-Erfolg	150	4,19	0,93	**78,0 %**	17,3 %	4,7 %
Kriterien für Reha-Erfolg operationalisieren	150	4,19	1,0	**74,7 %**	20,0 %	5,3 %
Zielgruppenspezifische Outcomes	150	3,70	4,05	**66,0 %**	24,0 %	10,05
Finden von Prädiktoren für den Reha-Erfolg	139	3,45	1,18	**50,4 %**	29,5 %	20,1 %
Nachhaltigkeit						
…der beruflichen Teilhabe	149	4,52	0,65	**91,3 %**	8,7 %	0,0 %
…des Gesundheitszustandes	149	4,32	0,74	**86,6 %**	12,1 %	1,3 %
…der Lebensqualität	149	4,32	0,88	**85,2 %**	11,4 %	3,4 %
…der Selbstständigkeit im Alltag	149	4,29	0,88	**83,9 %**	12,1 %	4,0 %
...der sozialen Teilhabe	149	4,24	0,84	**83,9 %**	13,4 %	2,7 %

Legende: n= Anzahl; MW= Mittelwert; SD= Standardabweichung (durchschnittliche Streuung um den Mittelwert);
Priorität hoch = Antwort 4 + 5; Priorität mittel = Antwort 3; Priorität niedrig = Antwort 1+2

Thieme

Tabelle S5: Prioritätseinschätzungen im Forschungsbereich Institution

Forschungsbereich Institution	n	MW	SD	Priorität hoch in %	Priorität mittel in %	Priorität niedrig in %
**Versorgungssystem**						
Entwicklung von zielgruppenspezifischen Angeboten						
… zur Sekundärprävention	148	4,33	0,88	**83,8 %**	11,5 %	4,7 %
… zur Tertiärprävention	148	4,19	0,84	**81,8 %**	14,9 %	3,4 %
… für zukünftige demografische Entwicklungen	148	4,30	0,91	**79,7 %**	16,2 %	4,1 %
… zur Primärprävention	148	4,25	1,11	**79,7 %**	9,5 %	10,8 %
… in Form von Nachsorgeangeboten	148	4,14	0,99	**77,7 %**	16,2 %	6,1 %
… zur Eltern-Kind-Rehabilitation	142	3,93	1,06	**70,4 %**	19,0 %	10,6 %
… für mobile Rehabilitation	142	3,85	1,06	**67,6 %**	20,4 %	12,0 %
… für Online Rehabilitation (Telerehabilitation)	148	3,90	1,19	**67,6 %**	16,9 %	15,5 %
… zur Prehabilitation	142	3,82	1,11	**66,2 %**	19,0 %	14,8 %
… zur aktiven Gesundheitsvorsorge	142	3,78	1,21	**63,4 %**	20,4 %	16,2 %
… in Form von digitalen Gesundheitsanwendungen	148	3,75	1,07	**62,8 %**	23,6 %	13,5 %
… in Form von therapeutischen Videospielen	141	3,38	1,15	**51,8 %**	26,2 %	22,0 %
… in Form von virtual/augmented reality-basierter Reha	141	3,28	1,30	**47,5 %**	24,1 %	28,4 %
Versorgungskontinuität						
Schnittstellenmanagement (Optimierung von Versorgungstrukturen)	148	4,29	0,87	**80,4 %**	17,6 %	2,0 %
Versorgungskontinuität im Sozialversicherungssystem	148	4,20	1,00	**77,7 %**	15,5 %	6,8 %
Behandlungspfade im Reha-System	148	4,06	0,96	**73,0 %**	22,3 %	4,7 %
Gesundheitsökonomie						
Gesundheitsökonomische Evaluationen von Reha-Programmen	146	3,97	0,94	**67,8 %**	26,7 %	5,5 %
Gesundheitsökonomische Fragestellungen	147	3,95	0,96	**64,6 %**	29,9 %	5,4 %
**Organisationsspezifische Rahmenbedingungen**						
Kontinuierliche Weiterentwicklung von Angeboten						
Weiterentwicklung von Therapiestandards	148	4,29	0,79	**84,5 %**	12,8 %	2,7 %
Einsatz von mobilem Monitoring	148	3,83	1,02	**64,9 %**	25,0 %	10,1 %
Einsatz von robotergestützten Therapiegeräten	141	3,70	1,03	**61,7 %**	24,1 %	14,2 %
Evidenzbasierte Strategieentwicklung und Innovation						
Entwicklung von Innovationen aus Ergebnissen der Reha-Forschung	148	4,20	0,87	**77,0 %**	19,6 %	3,4 %
evidenzbasierte Strategieentwicklung	148	3,99	0,98	**70,9 %**	20,3 %	8,8 %
rechtliche Rahmenbedingungen	141	3,27	1,19	**42,6 %**	33,3 %	24,1 %
Wissenstransfer						
Transfer wissenschaftlicher Erkenntnisse in die Praxis	148	4,36	0,87	**83,8 %**	13,5 %	2,7 %
Interprofessionelle Zusammenarbeit						
Zusammenarbeit im interdisziplinären Reha-Team	148	4,35	0,90	**84,5 %**	10,8 %	4,7 %

Legende: n= Anzahl; MW= Mittelwert; SD= Standardabweichung (durchschnittliche Streuung um den Mittelwert);
Priorität hoch = Antwort 4 + 5; Priorität mittel = Antwort 3; Priorität niedrig = Antwort 1+2

Tabelle S6: Prioritätseinschätzungen im Forschungsbereich Interdisziplinäre Forschung

FORSCHUNGSBEREICH INTERDISZIPLINÄRE FORSCHUNG	n	MW	SD	Priorität hoch in %	Priorität mittel in %	Priorität niedrig in %
Forschungsansätze						
Evaluationen						
wissenschaftliche Begleitung der Implementierung neuer Maßnahmen	148	4,05	1,00	**75,0 %**	14,9 %	10,1 %
Evaluierung bestehender Heilverfahren als Grundlage zur Verbesserung	150	4,00	0,96	**69,3 %**	26,0 %	4,7 %
Partizipative Forschung						
Miteinbeziehen der Praktiker*innen	144	4,22	1,04	**78,5 %**	13,9 %	7,6 %
Miteinbeziehen der Rehabilitand*innen	144	4,10	1,09	**75,0 %**	16,7 %	8,3 %
Studiendesigns						
studienübergreifende Vergleiche von Ergebnissen	144	3,90	0,97	**64,6 %**	29,2 %	6,3 %
experimentelle Studien (randomisiert mit VG und KG)	144	3,90	1,13	**63,9 %**	23,6 %	12,5 %
explorative Studien (gegenstandserkundend)	139	3,25	1,04	**41,0 %**	38,8 %	20,1 %
quasi-experimentelle Studien (methodisch hergestellte experimentelle Bedingungen)	139	3,18	1,14	**37,4 %**	37,4 %	25,2 %
deskriptive Studien (populationsbeschreibend)	139	3,13	0,99	**34,5 %**	43,9 %	21,6 %
Theorien und Modelle						
Überprüfung, Modifikation und Entwicklung						
Überprüfung etablierter Theorien und Modelle	146	3,75	1,04	**61,6 %**	28,1 %	10,3 %
Analyse von komplexen Interventionen ("Programmtheorie")	144	3,72	1,06	**60,4 %**	28,5 %	11,1 %
Implementierung von Klassifikationssystemen						
Implementierung und Nutzung von Klassifikationssystemen	145	3,85	1,10	**63,4 %**	24,8 %	11,7 %
Instrumente und Datennutzung						
Entwicklung und Validierung von Assessmentinstrumenten						
Testen der Eignung von Assessmentinstrumenten	146	3,84	0,98	**66,4 %**	24,7 %	8,9 %
Nutzbarmachung von vorhandenen Routinedaten						
Nutzbarmachen von vorhandenen personenbezogenen Routinedaten	146	4,06	0,99	**75,3 %**	17,1 %	7,5 %
Nutzen von Sekundärdaten (Gesundheitsregister)	146	3,75	0,99	**61,0 %**	29,5 %	9,6 %

Legende: n= Anzahl; MW= Mittelwert; SD= Standardabweichung (durchschnittliche Streuung um den Mittelwert);
Priorität hoch = Antwort 4 + 5; Priorität mittel = Antwort 3; Priorität niedrig = Antwort 1+2
